# Combination therapy with BYL719 and LEE011 is synergistic and causes a greater suppression of p-S6 in triple negative breast cancer

**DOI:** 10.1038/s41598-019-43429-7

**Published:** 2019-05-17

**Authors:** Yuan Yuan, Wei Wen, Susan E. Yost, Quanhua Xing, Jin Yan, Ernest S. Han, Joanne Mortimer, John H. Yim

**Affiliations:** 1 Department of Medical Oncology & Molecular Therapeutics, City of Hope Comprehensive Cancer Center and Beckman Research Institute, Duarte, CA USA; 2 Division of Surgical Oncology, City of Hope Comprehensive Cancer Center and Beckman Research Institute, Duarte, CA USA; 3 Division of Gynecologic Oncology, City of Hope Comprehensive Cancer Center and Beckman Research Institute, Duarte, CA USA

**Keywords:** Targeted therapies, Growth factor signalling

## Abstract

A third of patients with triple negative breast cancer (TNBC) have relapsed disease within 2–5 years from initial diagnosis, leaving an unmet need for therapeutic targets. TNBC frequently harbors alterations of the PI3K/AKT/mTOR pathway, but single agent PI3K/AKT/mTOR inhibitors have not shown marked efficacy. In this study, we investigated a strategy to improve efficacy of PI3K-α inhibitor BYL719 (alpelisib) in TNBC. While BYL719 is effective at inhibiting cell proliferation in T47D, a triple positive cell line, it had limited activity in TNBC. This may be partially due to persistent phosphorylation of RB, and incomplete inhibition of p-S6 in TNBC, since the inhibitory effect of BYL719 on p-RB and p-S6 was significantly reduced in TNBC compared to T47D cells. Addition of the CDK4/6 inhibitor LEE011 to BYL719 caused a simultaneous reduction of p-RB and p-S6, and a more complete inhibition of p-S6, leading to decreased expression of the pro-survival protein MCL-1, an induction of apoptosis, and an enhanced reduction of tumor growth in a PDX model of TNBC. These findings suggest that inhibition of p-RB and p-S6 is important for an effective response to the treatment of TNBC, and provides a strong rationale for clinical development of combination therapy with BYL719 and LEE011 for treatment of metastatic TNBC with intact RB.

Presentation: This study was presented in part as an abstract at the 2016 San Antonio Breast Cancer Symposium (P3-03-15) and the 2018 Cancer Research and Targeted Therapy in London.

## Introduction

Triple negative breast cancer is an aggressive subtype of breast cancer with limited treatment options and very poor prognosis for metastatic disease. Median progression-free survival with chemotherapy ranges from 2.5 to 4 months, and an average overall survival of 13 months in the metastatic setting^1,2^. The disproportionately high rate of mortality compared with hormone receptor positive breast cancer or HER2 positive breast cancer is largely due to the lack of effective targeted therapies, with the exception of tumors with germline BRCA1/2 mutation^3^. Early trials using the VEGF inhibitor^4^, c-Kit inhibitor^5^, EGFR inhibitor^6,7^, and histone deacetylase inhibitor^8^ failed to show efficacy. Androgen receptor (AR) targeted therapies such as bicalutamide and enzalutamide showed limited efficacy in the subpopulation of AR+ TNBCs^9,10^. Recent clinical trial data shows the potential utility of immune check point inhibitors; however, single agent response rates are modest, ranging between 5–19%^11–14^. These results highlight the unmet need to develop effective targeted therapy combinations for treatment of metastatic TNBC.

TNBC is a heterogeneous disease with at least 7 subtypes identified through mRNA expression profiling by the Vanderbilt Classification^15^: basal like-1 (BL-1); basal like-2 (BL-2); mesenchymal (M); mesenchymal stem-like (MSL); immunomodulatory (IM); luminal androgen receptor (LAR) and unstable (UNS) subtypes. The complexities of TNBC at the genomic level predict no single treatment approach will produce universal benefit across all subtypes, and combination therapy is likely to yield a better outcome. Individualized therapeutic approaches are needed based on the subtypes; however, the clinical utility of the molecular classifiers in guiding treatment decisions is still unclear.

The Cancer Genome Atlas (TCGA) analysis demonstrated that the most frequent tumor genomic alterations in TNBC involve genes associated with DNA damage repair, phosphatidylinositol 3-kinase (PI3K) signaling pathway^16^, and RAS/RAF/MEK pathways. The PIK3A-AKT-mTOR pathway alteration occurs in 40% of TNBCs. Non-basal subtypes (LAR, M and MSL) have demonstrated relatively high PIK3CA –activating mutations, and exhibit sensitivity to PI3K inhibitors *in vitro*^17^. Nevertheless, use of PI3K inhibitors as single agent therapy has proven minimally effective due to multiple feedback mechanisms^18^. Combination therapies with chemotherapy agents have shown synergy but are associated with more toxicities^19,20^. Recently, combination of PI3K inhibitor with CDK4/6 inhibitor has been shown to have a synergistic effect in tumor suppression in ER positive breast cancer^21,22^. Dysregulation of multiple CDK family members occurs commonly in human cancer; in particular, the cyclin D-CDK4/6-retinoblastoma protein (RB)-INK4 axis is universally disrupted, facilitating cancer cell proliferation^23,24^. CDK4/6 inhibitors (palbociclib, ribociclib and abemaciclib) in combination with hormone therapies are currently approved by the FDA to treat patients with estrogen receptor positive (ER+) and HER 2 negative breast cancers. Palbociclib has substantial activity in breast cancer cell lines of the LAR sub-type of TNBC, with similar levels of activity to ER positive (ER+) breast cancer cell lines^25^.

The current study was designed to investigate the efficacy of the PI3K-α inhibitor BYL719 (alpelisib), either alone or in combination with the FDA approved CDK4/6 inhibitor LEE011 (ribociclib) in TNBC. BYL719 is an α-isoform selective PI3K inhibitor, which has demonstrated clinical activity as a single agent and in combination with hormone therapy in patients with advanced HR+ BC. We hypothesize that combining PI3K-α inhibitor BYL719 and CDK4/6 inhibitor LEE011 may overcome single agent resistance, and provide synergistic suppression in TNBC.

## Results

### Effects of single agent BYL719 and LEE011 on cell viability of TNBC cell lines

The anti-tumor activity of single agent BYL719 and LEE011 was tested in TNBC cell lines representative of each molecular subtype through cell viability analysis. As shown in Table 1, the concentration that gave 50% inhibition (IC_50_) of cell viability for single agent BYL719 ranged from 3.68 μM to 26 μM. The IC_50_ for LEE011 was 13.1 μM in MDA-MB-231 cells, and was >40 μM in most other cells. These IC_50_ values for BYL719 were higher than the IC_50_ for T47D cell, a triple positive cell line, which is sensitive to BYL719.

**Table 1:** IC_50_ for BYL719, either alone or in combination, in TNBC^15^.

Cell line	Subtype	RB status	Mutations	Single Agent		Combination BYL719 + LEE011	
				BYL719 IC_50_ (μM)	LEE011 IC_50_ (μM)	BYL719 IC_50_ (μM)	IC_50_ Fold Reduction
HCC38	BL1	WT	CDKN2A; TP53	26.01	>40	11.88	2.19
MDA-MB-468	BL1	Mut	PTEN;SMAD4;TP53	11.28	>40	13.46	0.84
HCC1806	BL2	WT	CDKN2A;TP53;UTX	8.18	>40	5.75	1.42
HCC1187	IM	WT	TP53;CTNNA1;DDX18;HUWE1;NFKBIA	14.41	>10	11.04	1.30
BT549	M	Mut	PTEN;TP53	17.37	>40	14.61	1.19
Hs578T	MSL	WT	CDKN2A;TP53	19.84	>40	8.99	2.21
MDA-MB-231	MSL	WT	BRAF;CDKN2A;KRAS;NF2;TP53;PDGFRA	19.29	13.01	6.80	2.83
MFM223	LAR	WT	PIK3CA;TP53	3.68	>20	0.79	4.66
T47D	ER+/PR+/HER2+	WT	PIK3CA;TP53	0.26	25.24		

### Effects of combining BYL719 and LEE011 on signaling pathways

**Figure 1:**
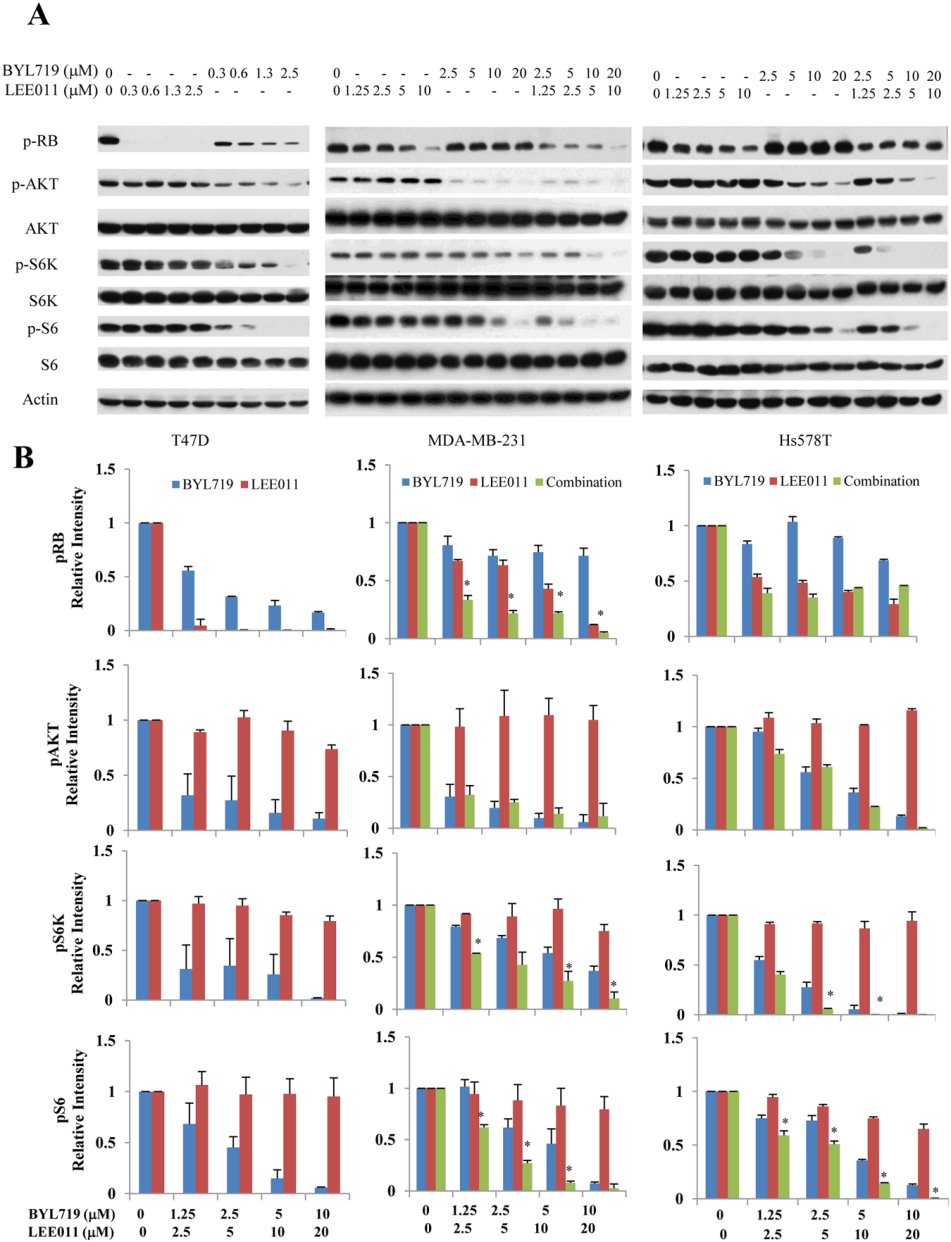
The combination of BYL719 and LEE011 leads to a simultaneous reduction of p-RB and p-S6 and a more complete inhibition of mTORC1 in TNBC cell lines. TNBC cells (MDA-MB-231 and Hs578T), as well as a triple positive cell (T47D), were treated with vehicle (DMSO), BYL719, LEE011, or BYL719 + LEE011 at various concentrations for 24 hours. (**A**) Whole-cell lysates were prepared and analyzed by Western blot for expression of signaling molecules. (**B**) Relative expression of p-AKT, p-S6K, and p-S6 was determined by measuring the density of each band and normalizing to the corresponding total protein. Relative expression of RB protein was normalized to β-actin since total RB protein was not detectable in these cells. Images are representative of results from three independent experiments. *P < 0.05, combination *vs*. vehicle, BYL719 or LEE011 alone. Data are mean ± S.D. from 3 experiments.

To understand the limited sensitivity of TNBC cell lines to BYL719 compared to T47D, the effect of BYL719 on the PI3K/AKT/mTOR pathway was analyzed in TNBCs. The inhibition of p-RB has previously been reported to be correlated with the sensitivity of cells to PI3K inhibitors^21^. As shown in Fig. 1, treatment with BYL719 alone not only significantly reduced the levels of p-AKT, p-S6K and p-S6, but also p-RB, in T47D cells. In contrast, treatment with BYL719 alone had little effect on p-RB in MDA-MB-231 and Hs578T cells. Although BYL719 was able to inhibit p-S6K and p-S6 in MDA-MB-231 and Hs578T cells, the inhibition was more significantly reduced in these two cells than in T47D cells, indicating that TNBC cells exhibit less inhibition of mTORC1 (as indicated by p-S6) than in sensitive cells, such as T47D. These findings are consistent with previous reports in other cancer cells that the sensitivity of cells to BYL719 may be dependent on the ability of BYL719 to suppress p-RB and mTORC1^21,26–28^.

Treatment with BYL719 alone led to the inhibition of the p-S6 pathway, but not the p-RB pathway in MDA-MB-231 and Hs578T cells. In contrast treatment with LEE011 alone led to inhibition of the p-RB pathway, but not the p-S6 pathway, in these two cell lines. However, the combination of LEE011 and BYL719 caused the suppression of both p-RB and p-S6K/p-S6 pathways, and a greater inhibition of p-S6K and p-S6 in MDA-MB-231 and Hs578T cells. Also, a stronger inhibition of p-RB was found in MDA-MB-231 cells treated with both BYL719 and LEE011. Taken together, these results demonstrate that the combined targeting of both PI3K and CDK4/6 pathways can inhibit multiple survival pathways, and results in a more complete inhibition of mTORC1.

### Synergistic effect of combined treatment with BYL719 and LEE011 on cell viability

**Figure 2:**
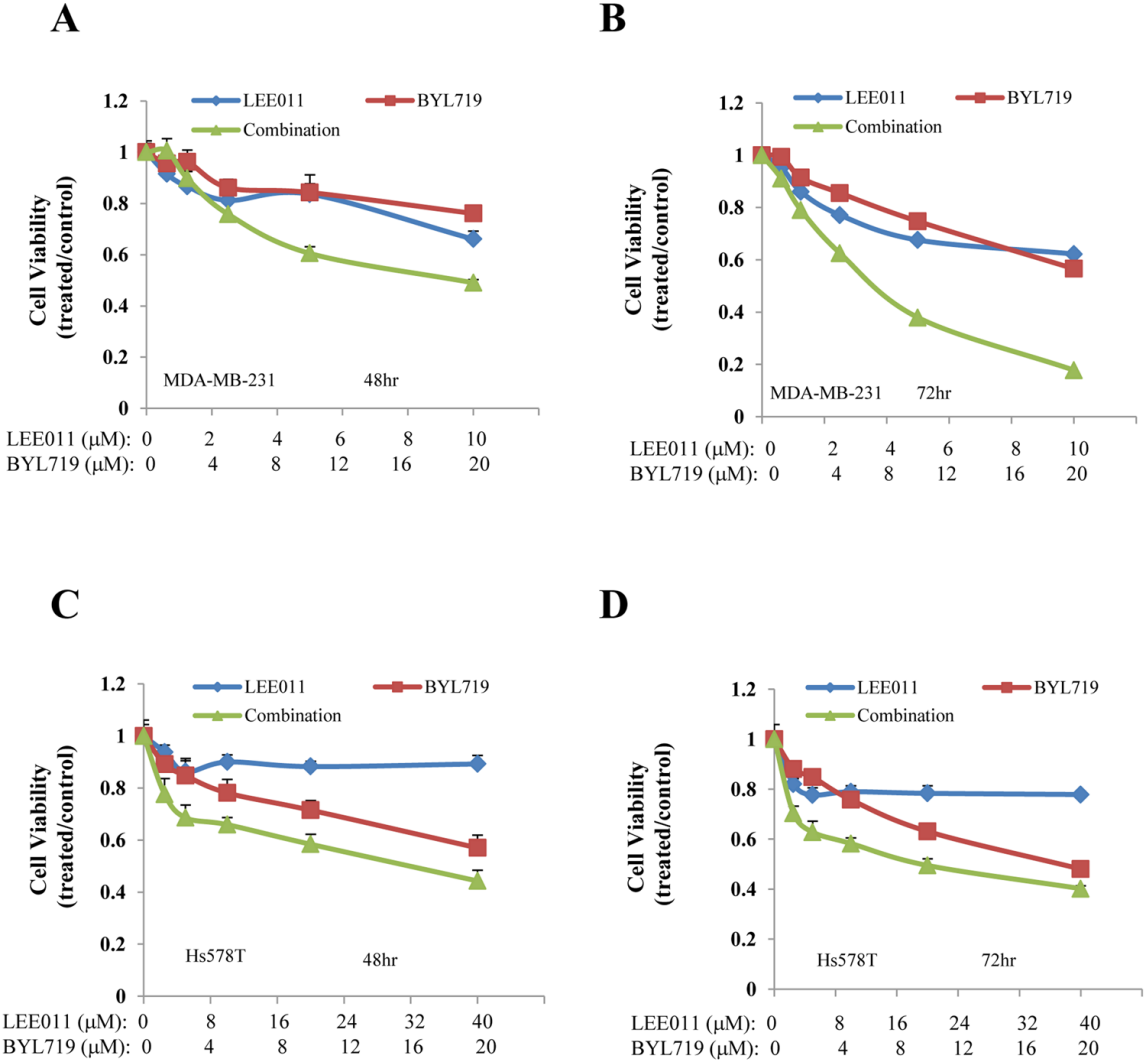
The combination of BYL719 and LEE011 decreases cell viability in TNBC cell lines. MDA-MB-231 (**A**,**B**) and Hs578T (**C**,**D**) cells were treated with BYL719 or LEE011, either alone or in combination, at various concentrations in a fixed molar ratio. Cell viability was determined after 48 hours (**A**,**C**) and after 72 hours (**B**,**D**). Data are mean of triplicates.

Given that BYL719 in combination with LEE011 led to a simultaneous inhibition of p-RB and p-AKT/p-S6K/p-S6, we next evaluated whether this combination resulted in reduced cell viability in TNBC cell lines. MDA-MB-231 and Hs578T cells were treated with BYL719 and LEE011 alone or in combination for 48 hours and 72 hours. As shown in Fig. 2, the combination treatment decreased cell viability much more robustly than either agent alone in both cell lines, with stronger activity seen at 72 hours.

To further understand whether this reduced cell viability could be reproduced in other TNBC cells, multiple TNBC cell lines representing several molecular subtypes were treated with BYL719 and LEE011 alone or in combination. As shown in Table 1, the concentrations of BYL719 that gave 50% inhibition (IC_50_) were significantly reduced by including LEE011 in the treatment of TNBC cell lines with intact RB (HCC38, HCC1806, HCC1187,Hs578T, MDA-MB-231 and MFM223), but not in cell lines with mutant RB (MDA-MB-468 and BT549). This reduced cell viability by combination treatment was observed in a number of representative cell lines, including BL1 (HCC38 and MDA-MB-468), BL2 (HCC1806), IM (HCC1187), M (BT549), MSL (MDA-MB-231 and Hs578T), and LAR (MFM223). It is important to note that in MFM223 (LAR TNBC subtype), the fold reduction of BYL719 IC_50_ induced by LEE011 was significantly higher (4.66 fold).

**Figure 3:**
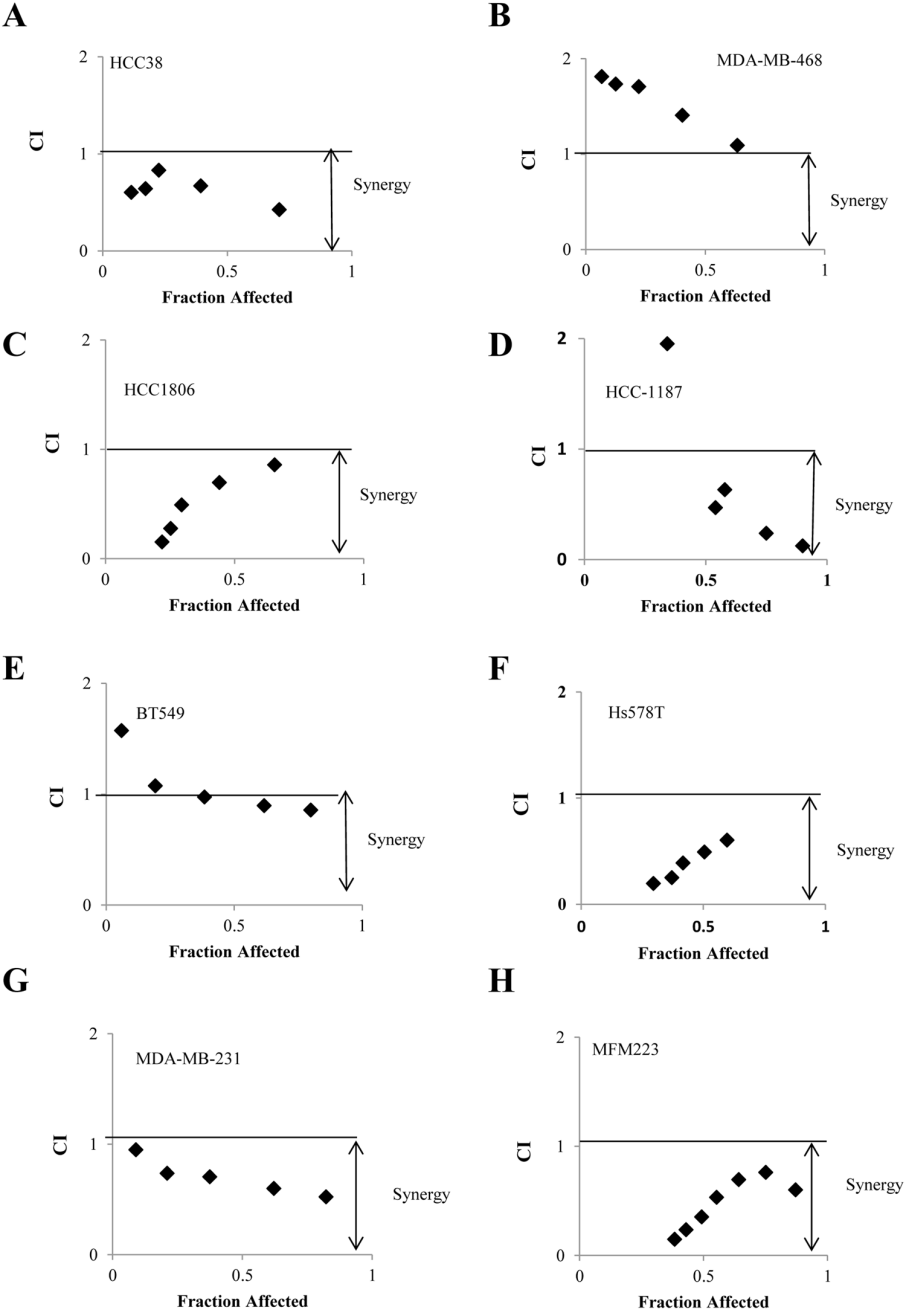
Synergistic interaction is observed between BYL719 and LEE011 in TNBC cells. (**A**–**H**) MDA-MB-231, Hs578T, HCC 1187, HCC38, HCC1806, MFM 223, BT548 and MDA-MB-468 cells were treated with BYL719 or LEE011 either alone or in combination at various concentrations. Cell viability was determined after 72 hours. The combination index (CI) was determined by Chou-Talalay method using the Calcusyn software.

To understand whether the increased activity was additive or synergistic, the Chou-Talalay method was used to determine the combination index (CI) (CI = 1, additive effect; CI < 1, synergism; CI > 1 antagonism)^29^. As shown in Fig. 3, the combination treatment produced strong to very strong synergism in a number of TNBC cell lines with intact RB (MDA-MB 231, HS578T, HCC-1187, HCC38, HCC-1806, MFM223), but not in TNBCs with mutant RB (BT 549 and MD-MB-468), providing evidence that this synergistic effect is associated with RB status.

### Effect of combined treatment with BYL719 and LEE011 on apoptosis

**Figure 4:**
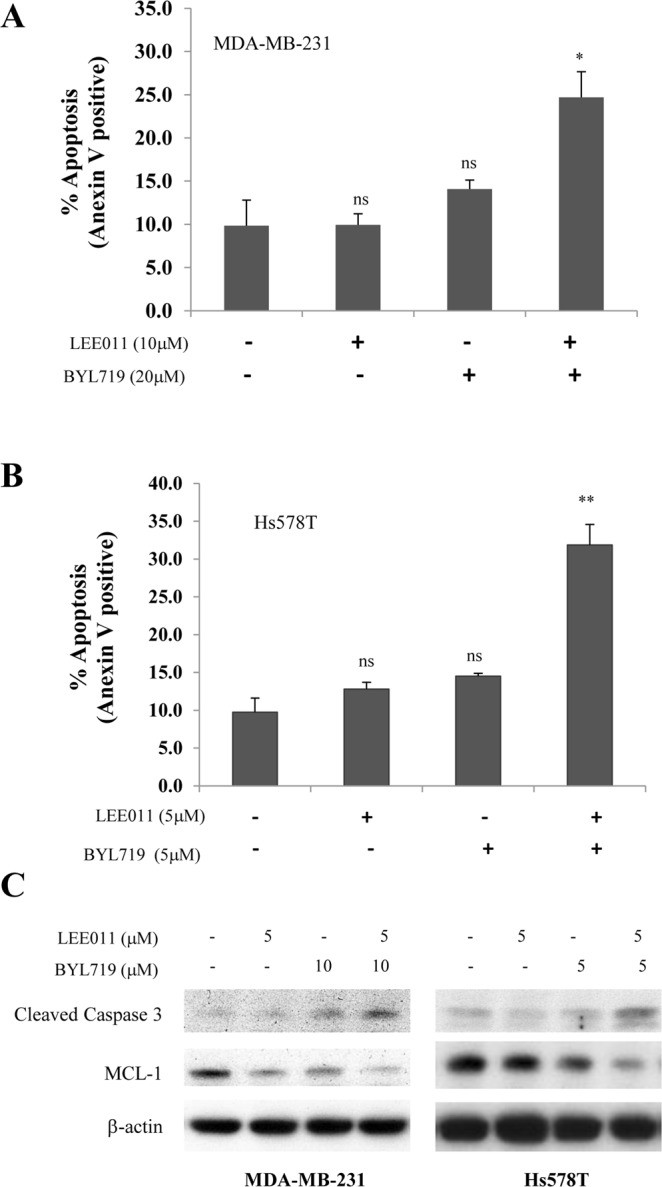
The combination of BYL719 and LEE011 induces apoptosis in MDA-MB-231 and Hs578T cells. Cells were treated with BYL719 and LEE011, either alone or in combination for 48 hours. Apoptosis was determined by (**A**,**B**) flow cytometry using Annexin V and PI staining or (**C**) Western blot analysis of cleaved caspase-3 and MCL-1. *P < 0.05, **P < 0.005, combination *vs*. vehicle, BYL719 or LEE011 alone. NS: not significant, BYL719 or LEE011 *vs*. vehicle. Data represents the mean ± SD of three preparations.

To determine whether the reduced cell viability could be due to the induction of apoptosis, cells were treated with BYL719 and LEE011 alone or in combination for 48 hours, and the number of apoptotic cells was determined by Annexin V staining. As shown in Fig. 4, BYL719-induced apoptosis increased from 4.27% to 20.39% when combined with LEE011 in MDA-MB-231 cells (Fig. 4A), and from 14.5% to 31.9% in Hs578T cells (Fig. 4B). Consistent with the Annexin V staining results, the increase of cleaved caspase 3, an apoptotic marker, and reduction of MCL-1, an anti-apoptotic protein, were also found in the cells that were treated with both BYL719 and LEE011 (Fig. 4C). These results indicate that inhibition of both PI3K and CDK4/6 pathways effectively suppresses cell survival in TNBC cells by promoting apoptosis.

### Anti-tumor activities of BYL719 and LEE011 in PDX models of human TNBC

**Figure 5:**
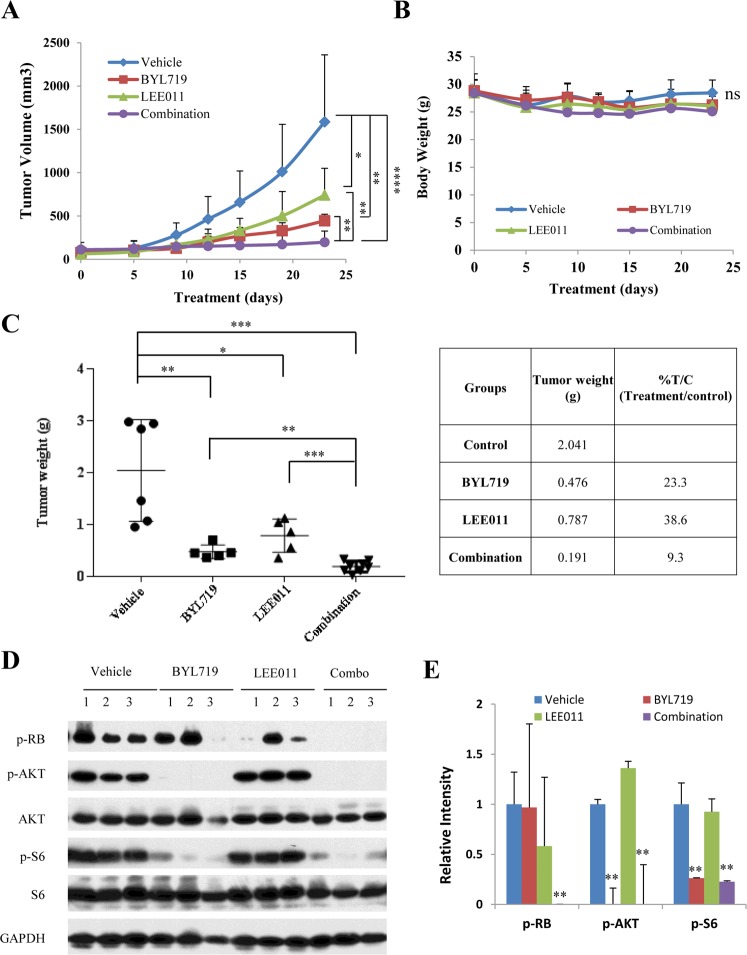
The combination of BYL719 and LEE011 induces anti-tumor activity in a TNBC PDX model. PDX tumors were surgically implanted into mammary fat pad of 6- to 8- week-old female NSG mice. Mice were treated daily by oral gavage with vehicle, BYL719 (30 mg/kg), LEE011 (75 mg/kg) or combination of both. (**A–C**) Tumor volume and body weight were measured 1–2 times per week, and tumor weight was measured at the end of the treatment. (**D**) Effect of BYL719 and LEE011 on the expression of signaling molecules in tumors was analyzed by Western blot. Whole-cell tumor lysates were prepared and analyzed by Western blot for expression of phosphorylated RB, AKT, and S6. (**E**) Relative expression of p-RB, p-AKT, and p-S6 was determined by measuring the density of each band and normalizing to GAPDH. Data represents the mean ± SD (n = 5–10). *P < 0.05; **P < 0.005; ***P < 0.0005, ****P < 0.0001, ns, not significant.

To test the treatment effect of the combination therapy of BYL719 and LEE011, a PDX model of chemotherapy-resistant TNBC was used. Exome sequencing of the tumor identified the following genomic alterations: PIK3CA E542K mutation, PTEN loss, CDK4 amplification, TP53 H179R mutation, KIT amplification, PDGFRA amplification, KDR amplification, and NOTCH2 exon 3 truncation. The combination of BYL719 and LEE011 showed synergistic tumor suppression, which was statistically significant compared to either agent alone (Fig. 5A). No toxicity was observed in mice with any of the treatments, whether the drugs were used alone or in combination, as indicated by absence of significant (>5%) change in body weight (Fig. 5B). Tumor weight at the end of treatment showed significant reduction in the combination therapy compared to single agents (Fig. 5C). Tumor weight decreased from 2.041 g to 0.476 g with BYL719 alone at a daily dose of 30 mg/kg, and from 2.041 g to 0.787 g with LEE011 alone at a daily dose 75 mg/kg. The combination treatment further decreased the tumor weight to 0.191 g.

To examine the effect of therapy on the downstream molecular targets, tumor tissue lysates were analyzed for the expression of p-RB, p-AKT, AKT, p-S6 and S6 by Western blot analysis. As shown in Fig. 5D,E, the combination of BYL719 with LEE011 led to the suppression of both p-RB and mTORC1 pathways and a greater inhibition of p-RB, which was consistent with the *in vitro* results shown in Fig. 1. Overall, these results support a consistent synergistic effect of PI3K-α and CDK4/6 inhibition in TNBC *in vitro* and *in vivo* in RB-intact tumors.

## Discussion

A third of patients with TNBC have relapsed disease within 2–5 years from initial diagnosis. This highlights the unmet need to develop effective targeted therapies in this population^2,30^. TNBC frequently harbors alterations of the PI3K/AKT/mTOR pathway, but single agent PI3K/AKT/mTOR inhibitors are not effective^31^. In this study, we showed that dual-targeting of PI3K-α and CDK4/6 provided synergistic suppression of TNBC cell lines and a PDX mouse model in an RB-dependent and subtype-independent manner. We found that the limited activity of BYL719 in TNBC may be partially due to persistent phosphorylation of RB and incomplete inhibition of the PI3K/AKT/mTOR pathway (as indicated by p-S6) in TNBC. Addition of the CDK4/6 inhibitor LEE011 to BYL719 caused a simultaneous reduction of p-RB and p-S6, and a more complete inhibition of mTORC1. This led to the decreased expression of pro-survival protein MCL-1, a synergistic inhibition of cell survival, and the reduction of tumor growth in this PDX model of TNBC.

The cyclinD:CDK4/6:RB axis is dysregulated in a variety of human cancers^24,32–34^. Targeting this pathway has proven to be a successful therapeutic approach in ER+ breast cancer with three CDK4/6 inhibitors currently used in the clinic^35^. CDK4/6 inhibitors halt cell cycle progression and induce G1 cell cycle arrest^33^. The CDK4/6 inhibitor LEE011 (ribociclib) has been approved for treatment of ER+ metastatic breast cancer. It is believed that intact RB is required in order for a CDK4/6 inhibitor to be effective in ER+ breast cancer, but cyclin D1 overexpression or loss of p16^INK4A^ did not predict response to palbociclib in the PALOMA-1 study^36^. Loss of RB expression has been reported in 20–30% of total breast cancers and approximately 40% of TNBCs^37^. O’Brien *et al*. identified RB-dependence of abemaciclib in multiple TNBC cell lines^38^. TNBC cell lines with high baseline total and phosphorylated p-RB, accompanied by low P16 level are among the most sensitive to abemaciclib^38^. Consistent with this report, our study has shown that CDK4/6 inhibition can sensitize TNBC cancer cells to PI3K inhibition, producing a greater reduction of cell viability in an RB-dependent and subtype-independent manner^39,40^.

Although our results suggest a possibility that the synergistic effect by combining BYL719 and LEE011 is associated with RB status, the two cell lines with RB mutated in our study that did not have synergistic effect (MDA-MB-468 and BT549) are also PTEN-deficient lines. PTEN is a tumor suppressor that de-phosphorylates phosphatidylinositol (3,4,5)-triphosphate (PIP), reversing the activation of PI3-kinase. PTEN also functions independently of its phosphatase in the nucleus, where it mediates cell proliferation, genome stabilization, and regulation of DNA repair^41^. PTEN deficient cell lines have been previous shown to depend more on p110-β instead of p110-α^42–45^. In comparison, both the MFM223 cell line and the PDX model have PIK3CA mutations that likely depend on p110α, and respond well to the single agent BYL719 treatment. Recent clinical trials suggested that many PTEN-deficient tumors don’t respond to PI3K inhibitors^46^. It is possible that the pan-PI3K inhibitors currently in trials are less effective on p110β compared to p110α; however, it is also possible that PTEN loss activates more than the PI3K pathway. PTEN loss may be more resistant to PI3K inhibition than tumors driven by p110α activation. Further study is needed to investigate the therapeutic potential of p110β-specific inhibitors in combination of CDK4/6 inhibitors for the treatment of PTEN-deficient TNBC.

The capability of the CDK4/6 inhibitor to increase the activity of PI3K was previously shown in TNBC carrying PIK3CA mutations treated with the CDK 4/6 inhibitor palbociclib and the pan-PI3K inhibitor taselisib^47^. Teo *et al*. also showed that dual blockade of PI3Kα and CDK4/6 is synergistic and immunogenic in multiple RB-wildtype TNBC models^48^. Our current finding is consistent with these previous reports. In addition, we demonstrated that combined inhibition of CDK4/6 and PI3Kα caused a simultaneous reduction of p-RB and p-S6 and a more complete inhibition of the PI3K/AKT/mTOR pathway, leading to a decreased expression of pro-survival protein MCL-1 and the induction of apoptosis. These results suggest that p-RB and p-S6 may act synergistically to control cancer cell survival and suppression of both pathways may be needed for an optimal anti-tumor activity of BYL719 in TNBCs. Furthermore, our results agree with previous findings that complete inhibition of p-S6 is important for sensitivity of PI3K inhibition in melanoma cells and ER+ breast cancer cells^21,26–28^. In addition to breast cancer, the synergistic anti-tumor effect of dual inhibition of the PI3K/AKT/mTOR pathway and CDK4/6 has also been demonstrated in malignant pleural mesothelioma, T-cell acute lymphoblastic leukemia, and mantle cell lymphomas^49–51^.

Other co-targeting strategies such as combining CDK4/6 inhibitors with chemotherapy have been tested. Palbociclib was found to antagonize the cytotoxic effect of doxorubicin and taxanes in the RB-intact TNBC cell lines MDA-MB-231 and Hs578T^52,53^. Increasing evidence suggests that the timing of palbociclib and paclitaxel use is critical. Increased paclitaxel cytotoxicity was found when cells were pre-exposed to palbociclib for synchronization^53^. A phase I trial of palbociclib and paclitaxel showed safety and early efficacy^54^. CDK4/6 inhibition with abemaciclib combined with anti-mitotic agents, both *in vitro* and *in vivo*, did not antagonize the effects of either agent. Co-administration of docetaxel with either palbociclib or ribociclib after CDK4/6-inhibitor pre-treatment blocked apoptosis induction, whereas co-treatment with abemaciclib did not^38^.

Asghar *et al*. has identified that TNBC cell lines of the luminal-androgen receptor (LAR) and mesenchymal-stem like (MSL) subsets were sensitive to palbociclib, and sensitivity was associated with the expression of androgen receptor and the absence or low levels of cyclin E1^47^. They also found that PI3K inhibition was synergistic with palbociclib in PIK3CA-mutated TNBC cell lines, with a greater effect in LAR/MSL subgroups compared to M/basal subgroups. LAR cell lines represent a TNBC subgroup that may benefit from CDK4/6 inhibition. This is consistent with our current results, that adding LEE011 to BYL719 leads to a significant 4.66 fold reduction of BYL719 IC_50_. This confirms the significant efficacy of CDK4/6 inhibitor in combination with PI3K-α inhibition in the LAR subtype of TNBC. TNBC subtype-dependent inhibition was not clearly demonstrated in the other cell lines.

In our study, synergy of BYL719 and LEE011 was observed in a PDX model of TNBC with genomic mutations in both the PI3K and CDK4/6 pathways: PIK3CA (E542K), PTEN loss, CDK4 amplification, TP53 (H179R), *KIT* amplification, *PDGFRA* amplification, *KDR* amplification, and *NOTCH2* truncation exon 3. Whether tumors that harbor these mutations are necessary to elicit a response to the combination is currently unknown. Zhang *et al*. developed a panel of PDX models representing multiple TNBC subtypes (IM, BL1, BL2, and M) in order to test the preclinical drug efficacy of mTOR inhibitors^55^. Ideally PDX models representing each subtype should be used to verify the efficacy of combination BYL719 and LEE011 treatment.

## Conclusion

This study demonstrates the synergistic effect of dual-targeting of the PI3K-α and CDK4/6 pathways in RB-intact TNBC. Given the data from the current study, as well as other published work, there is a strong rationale for clinical development of combination therapy with BYL719 and LEE011 for treatment of metastatic TNBC with intact RB. The synergistic effect is particularly notable in the LAR subtype of TNBC, and further studies are needed to address the significance of this result.

## Materials and Methods

### Cell lines, cell culture, and reagents

The human breast cancer cell lines used in this analysis, including T47D, MDA-MB-468, BT549, HCC38, HCC1187, and Hs578T were obtained from American Type Culture Collection (Rockville, MD). MDA-MB-231 was kindly provided by Dr. Emily Wang (City of Hope). BT549, HCC38 and HCC1187 were cultured in RPMI 1640 medium (Mediatech Inc., Manassas, VA) supplemented with 10% fetal bovine serum (FBS, Atlanta Biologicals, Norcross, GA) and 1% penicillin/streptomycin. MDA-MB-468 cells were propagated in 1:1 DMEM/F12 (1:1) (Gibco, Invitrogen) supplemented with 10% FBS and 1% penicillin/streptomycin. MDA-MB-231 and Hs578T were cultured in DMEM medium (Mediatech Inc., Manassas, VA) supplemented with10% fetal bovine serum and 1% penicillin/streptomycin.

All cells were incubated at 37 °C at 5% CO_2_. BYL719 (alpelisib, selective PI3K-α inhibitor) and LEE011 (ribociclib, highly specific CDK4/6 inhibitor) were obtained under a Material Transfer Agreement with Novartis (Basel, Switzerland).

### Proliferation assays and multiple drug effects analysis

Cells (4000 per well) were plated in 96-well plate format in 100 μl growth medium. Cells were incubated with DMSO or increasing concentrations of BYL719 and LEE011, and cell viability was determined 72 hours later. The following starting doses were used: MDA-MB-468, HCC1187, and MDA-MB-231: (1.25 μM BYL719 and 0.625 μM LEE011); BT549 (2.5 μM BYL719 and 2.5 μM LEE011); HCC38 and Hs578T (1.25 μM BYL719 and 2.5 μM LEE011). Viable cells were determined by the MTT assay according to manufacturer’s instruction (Promega, Madison, WI, USA). Briefly, after treatment, the media was removed and 3-(4,5-dimethyl-thiazol-2-yl)-2,5-diphenyltetrazolium bromide (MTT) dye was added to each well and incubated for 4 hours. The formazan crystals were dissolved in dimethyl sulfoxide (DMSO) after removing the media. Absorbance was read at 570 nm. The IC_50_ was determined using the CalcuSyn software (Biosoft, Ferguson, MO). The combination index (CI) was determined by Chou-Talalay method using CalcuSyn software (Biosoft, MO)^29^.

A CI < 1 indicates synergy, CI > 1 indicates antagonistic interactions, and a CI value = 1 indicates additive effects.

### Western blot analysis

Cells were grown in complete medium overnight and treated with DMSO or drugs at recorded concentrations and times. Cells were lysed in RIPA lysis buffer containing Halt protease and phosphatase inhibitors (Thermo Scientific Inc.). Equal amounts of protein were separated by SDS-polyacrylamide gel electrophoresis, transferred to polyvinylidene fluoride membranes and incubated with total and phosphorylated protein-specific antibodies. Antibodies against p-RB (catalog no. 8180; 1:800 dilution), p-AKT (S473) (catalog no. 9271; 1;1000 dilution), AKT (catalog no. 9272; 1;1000 dilution), p-S6K1 (catalog no. 9234; 1;1000 dilution), S6K1 (catalog no. 2708; 1;1000 dilution), p-S6 (catalog no. 2211; 1;3000 dilution), S6 (catalog no. 2217; 1;3000 dilution), and β-actin (catalog no. 4970; 1;3000 dilution) were obtained from Cell Signaling Technology (Danvers, MA). Binding of the primary antibody was detected using a horseradish peroxidase (HRP)-conjugated secondary antibody and chemiluminescent substrates (Thermo Scientific Inc.). Signal was detected by exposing standard X-ray film. Films were scanned using Epson Perfection V750 pro Scanner. The density of each protein band was quantified by Image J software (NIH). The density of phosphoprotein was normalized to the corresponding total proteins.

### Annexin V staining

Annexin V apoptosis detection kit (BD bioscience) was used to measure apoptosis. Breast cancer cells were treated with BYL719, LEE011, or both at various concentrations for 48 hours. All cells, including floating and attached cells, were collected and stained with FITC-Annexin V and PI. The staining intensity was quantified using fluorescence-activated cell sorting (FACS).

### TNBC patient-derived xenograft (PDX) model

The combination of BYL719 and LEE011 was tested *in vivo* using a TNBC patient-derived xenograft (PDX) based upon the molecular profile of the tumor. After obtaining informed written patient consent, a triple negative breast tumor sample was obtained from the patient at the time of surgery at City of Hope under protocol approved by Institutional Review Board (IRB). All methods were performed in accordance with the relevant guidelines and regulations. Fresh primary tumor tissues (2-3 mm in diameter) were surgically implanted into mammary fat pad of 6- to 8- week-old female NOD/SCID/IL2Rgamma null (NSG) mice. Once the xenograft was established, the tumor was removed, cut into small fragments, surgically implanted into mammary fat pad of mice to expand the xenograft numbers. When the xenografts were palpable, animals were randomized into 4 groups and treated by oral gavage with vehicle (30% Solutol HS15 + 0.5% methycellulose, daily), BYL719 (30 mg/kg, daily), LEE011 (75 mg/kg, daily), or a combination of both agents. Tumor volumes were assessed using calipers 1-2 times per week and determined using the formula (width)^2^ × length × 0.52. Body weight was monitored weekly as an indicator of drug-induced toxicity and overall health of the mice. Tumor was harvested and measured for the weight at day 23 of the experiment. All animal studies were carried out under protocols approved by the Institutional Animal Care and Use Committee (IACUC) at City of Hope in accordance with all applicable federal, state, and local requirements and institutional guidelines.

### Statistical methods

Data are presented as mean ± S.D from 3 experiments. All the experiments were carried out in triplicate or more. Student’s t-test was used to compare the mean of two groups. ANOVA was used to compare the difference for the multiple groups. A value of p < 0.05 was considered statistically significant.
